# Response to Oxidative Stress in *Sporothrix schenckii*

**DOI:** 10.3390/jof11060440

**Published:** 2025-06-10

**Authors:** Estela Ruiz-Baca, Pablo Jaciel Adame-Soto, Carlos Antonio Alba-Fierro, Ana Lilia Martínez-Rocha, Armando Pérez-Torres, Angélica López-Rodríguez, Yolanda Romo-Lozano

**Affiliations:** 1 Facultad de Ciencias Químicas, Universidad Juárez del Estado de Durango, Av. Veterinaria S/N, Durango C.P. 34120, Mexico; pablo.adame@ujed.mx (P.J.A.-S.); carlos.alba@ujed.mx (C.A.A.-F.); angelica.lopez@ujed.mx (A.L.-R.); 2 Departamento de Biología, División de Ciencias Naturales y Exactas, Universidad de Guanajuato, Guanajuato C.P. 36050, Mexico; anamartinez@ugto.mx; 3 Facultad de Medicina, Universidad Nacional Autónoma de México, Ciudad de Mexico C.P. 04510, Mexico; armandop@unam.mx; 4 Centro de Ciencias Básicas, Universidad Autónoma de Aguascalientes, Av. Universidad No. 940, Aguascalientes C.P. 20131, Mexico

**Keywords:** *Sporothrix schenckii*, phagocytic cells, oxidative stress, reactive oxygen species

## Abstract

Oxidative stress is key in immune defense against fungal infections, such as those caused by *Sporothrix schenckii*, the dimorphic fungus responsible for sporotrichosis. Phagocytic cells utilize oxidative stress as a crucial mechanism to control pathogen spread. During *S. schenckii* infection, phagocytic cells recognize pathogen-associated molecular patterns (PAMPs) on their surface through conserved transmembrane or soluble receptors, known as pattern recognition receptors (PRRs). This recognition triggers a cascade of immune responses, including the generation reactive oxygen species (ROS) essential for pathogen elimination. However, *S. schenckii* has developed sophisticated mechanisms to evade and counteract this response, contributing to its persistence in the host. These mechanisms include the production of antioxidant enzymes, alterations to its cell wall (CW), and the production of melanin, which helps neutralize oxidative stress. In addition, *S. schenckii* modulates the production of other proteins, such as moonlighting proteins, suggested to have roles in immune evasion and stress response, helping its survival in the host. These strategies, along with the modulation of gene expression, allow the fungus to survive and persist inside the immune system’s hostile environment, facilitating the progression of the infection. Understanding these interactions between phagocytic cells and *S. schenckii* is key to developing more effective therapeutic strategies to combat sporotrichosis.

## 1. Introduction

Sporotrichosis, caused by the pathogenic species of the genus *Sporothrix,* is the most prevalent and widely distributed mycosis worldwide, being more frequent in Latin America [1,2]. It is a subacute or chronic subcutaneous mycosis of animals and humans. The infection is transmitted through traumatic inoculation via sapronotic, enzootic, or zoonotic routes [3,4]. The first anatomic site affected is the skin, subcutaneous tissues, and the adjacent lymphatic system. Other rare clinical forms may compromise joints, bones, the central nervous system, and lungs [5]. Cutaneous sporotrichosis can manifest as fixed, lymphocutaneous, or disseminated forms, with clinical presentations varying depending on the host immune response and the specific species involved [6]. This mycosis is a benign disease in most immunocompetent patients, but it has high morbidity and may be fatal in immunocompromised individuals [7].

The genus *Sporothrix* includes thermodimorphic fungi found in soil and decaying plant material. Once the infection has been transmitted, thermodimorphic transition is necessary to progress to the disease. During this process, the mycelial or saprophytic morphotype transforms into yeast, the parasitic morphotype [8]. The saprophytic morphotype grows into a filamentous form, which is characterized by slender, hyaline, and septate hyphae that produce hyaline or pigmented conidia and, during the parasitic phase in the host tissues, budding yeasts [9]. The most important species are *S. schenckii sensu stricto*, *S. luriei, S. albicans*, *S. brasiliensis*, *S. globosa*, *S. mexicana*, *S. pallida*, *S. brunneoviolacea*, and *S. dimorphospora,* being the most common species that cause sporotrichosis in humans, as well as *S. schenckii sensu stricto, S. brasiliensis, S. globosa, S. mexicana,* and *S. luriei* [10,11]. Other *Sporothrix* species primarily function as environmental saprophytes [11].

Pathogenic species of *Sporothrix* have several virulence factors, such as dimorphism and the production of melanin and other molecules, which contribute to fungal adhesion, invasion, and survival in mammalian hosts [12]. In *S. schenckii*, the virulence factors most relevant are cell wall proteins (CWPs), kinases, heat shock proteins, extracellular and intracellular proteinases, extracellular vesicles, lipids, the capacity to form biofilms, melanin, and the production of antioxidant enzymes [13,14,15].

During infections, the fungal pathogen must confront several mechanisms of defense deployed by the host to remove it. In the first encounter, the cells of the innate immune system, mainly epithelial cells, neutrophils, macrophages, monocytes, and dendritic cells (DC), recognize conserved molecular structures on the pathogen surface, known as pathogen-associated molecular patterns (PAMPs), via conserved transmembrane or soluble receptors, named pattern recognition receptors (PRRs). Some PRRs include Toll-like receptors (TLRs) and C-type lectin receptors. Upon engagement of PRRs with PAMPs, immune cells modulate the release of inflammatory cytokines, chemokines, and the activity of complement elements, as well as the formation of the NLRP3 inflammasome [16]. Moreover, neutrophils and macrophages can phagocytize fungal cells, triggering intracellular microbicidal events to eliminate the pathogen [17]. Macrophages can internalize both conidia and yeasts of *S. schenckii* [8], with complement participation only for the recognition of yeasts of *S. schenckii* [18], kill them intracellularly, and generate a proinflammatory response that activates other cells. Neutrophils possess various powerful oxidative and nonoxidative microbicidal compounds. Microbicidal mechanisms comprise intracellular oxidative burst, degranulation, the production of proinflammatory cytokines, and neutrophil extracellular traps (NETs) [19,20]. DCs are responsible for discriminating between different fungal morphotypes or growth stages and are important antigen-presenting cells that connect the innate and adaptive immune system [21]. Adaptive immunity, particularly Th1 and Th17 responses, plays a critical role in controlling fungal dissemination, whereas a shift toward Th2 responses has been associated with increased clinical severity [22].

Fungal cells are phagocytosed and located in phagolysosomes, an acidic compartment involved in phagocyte-degrading activities. Additionally, they produce enzymes that generate reactive oxygen species (ROS), reactive nitrogen species (RNS), and lytic enzymes. During this oxidative burst, the main ROS produced are hydrogen peroxide (H_2_O_2_), radical superoxide (O_2_⁻), and hypochlorous acid (HOCl) [23,24]. The oxidative burst promotes cell and tissue damage and a state of oxidative stress in the infected area, which affects the immune response locally, favoring the survival of the fungus in the host. Moreover, melanin production by *S. schenckii* [25] and its CWPs confer protection against ROS [20,24,25,26]. Additionally, resistance to some ROS, such as (H_2_O_2_), has also been shown to depend on some transcriptional factors [27].

The interplay between the host’s immune response and virulence factors of *Sporothrix* sp. during *S. schenckii* infection (sporotrichosis) is only partially understood [28]. The study of oxidative stress as an immune response must consider pro-oxidant and antioxidant enzymes, small-molecule antioxidant compounds, and gene transcription due to redox signal. Any imbalance between oxidants and antioxidants can induce molecular damage and inflammatory responses [29]. Also, it is imperative to consider the role that each plays in oxidative stress during the host–pathogen interaction. Antioxidant enzymes play the principal role in antioxidant defense, not just small-molecule antioxidant compounds. Despite advances in understanding these mechanisms, there are aspects to clarify regarding their regulation and their impact on fungal virulence. Furthermore, if the innate immune response is not efficient in eliminating fungi, the adaptive immune response will be activated [13]. This review analyzes the current knowledge of the oxidative stress response in *S. schenckii* and its contribution to pathogenicity and virulence in this fungus.

## 2. Phagocyte-Induced Oxidative Stress and Evasion Strategies of *S. schenckii* During Infection

**Figure 1:**
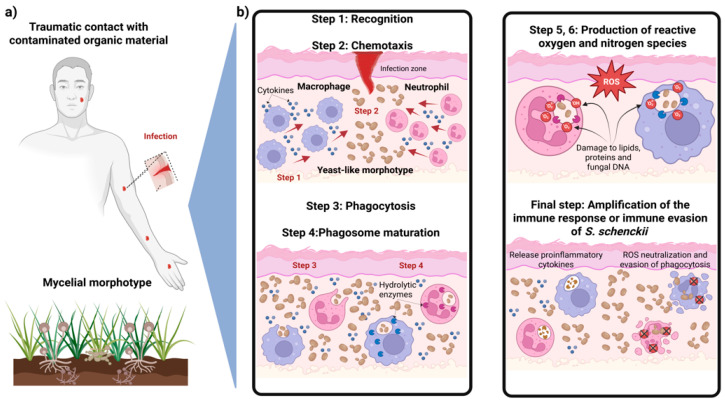
The role of phagocytic cells in the innate immune response to *S. schenckii* infection. (**a**) Host infection by the mycelial morphotype following trauma with contaminated material. (**b**) The innate immune response steps from pathogen recognition to intracellular events in phagocytic cells: recognition, chemotaxis, phagocytosis, phagosome maturation, ROS/RNS production, and either immune response amplification by phagocytic cells or the evasion mechanism used by *S. schenckii* to avoid phagocytic elimination. Note: Red arrows indicate the direction of immune cell signaling and activation.

The immune response to eliminate *S. schenckii* via phagocytic cells involves a coordinated series of steps (Figure 1). The first step is pathogen recognition, where macrophages and neutrophils recognize *S. schenckii* through PRRs which detect PAMPs on the fungal surface [30]. Recognition of *S. schenckii* activates intracellular signaling pathways that promote phagocytosis and the release of proinflammatory cytokines. The second step is chemotaxis and adhesion to recruit phagocytic cells to the infection site through chemokines such as IL-8 [31]. These cells adhere to the fungal pathogen through specialized receptors. The third step is phagocytosis of the pathogen by the phagocyte, forming an intracellular vesicle called the phagosome [32]. The fourth step is phagosome maturation, where host cells form a phagolysosome containing hydrolytic enzymes in an acidic environment with antimicrobial peptides, degrading fungal components [33,34]. The fifth and sixth steps involve the production of ROS and RNS, respectively, which directly damage fungal structures such as lipids, proteins, and DNA, thereby contributing to pathogen elimination [35,36]. The final step is cytokine release and amplification of the immune response. During the interaction with *S. schenckii*, phagocytic cells release proinflammatory cytokines such as TNF-α, IL-1β, and IL-12, which amplify the inflammatory response and recruit additional immune cells [28].

After *S. schenckii* infection, the fungus encounters hostile conditions within the host, generated by phagocytic cells of the immune system [25], leading to an oxidative stress environment and an imbalance between the production of ROS and the fungus’s ability to detoxify them. During sporotrichosis, macrophages and neutrophils produce large amounts of ROS, with various functions (Table 1), through the NADPH oxidase complex [37]. These enzymatic complexes transfer electrons to molecular oxygen to eliminate invading microorganisms [38,39].

Oxidative stress presents a constant challenge for *S. schenckii* due to its continuous interaction with ROS such as superoxide (O_2_⁻), which can later be dismutated into hydrogen peroxide (H_2_O_2_) and subsequently converted into hydroxyl radicals (OH), highly reactive species capable of causing severe damage to fungal cells [24,39,46]. Furthermore, the oxidizing environment could activate the inflammasome NLRP3 assembly and connect the innate immune response with the adaptive one [16,47].

Despite the immune system’s robust response to this fungal infection, *S. schenckii* possesses mechanisms to cope with oxidative stress, allowing it to neutralize ROS and evade phagocytic elimination (Table 2). Studies in *C. glabrata* report that evasion strategies are linked to genetic regulation, identifying genes involved in oxidative stress resistance whose expression increases in response to unfavorable conditions [48]. Using these defense mechanisms, *S. schenckii* can survive and replicate within phagocytes, particularly macrophages, using immune cells as a niche and later disseminating to other tissues [48,49]. These mechanisms enable *S. schenckii* to adapt and persist within the host, contributing to its pathogenicity [41,50].

Comparative studies identified *S. brasiliensis* and *S. schenckii* as the most pathogenic species within the *Sporothrix* complex [26,54]. Although they share some characteristics compared to other species in the complex, the pathogenic mechanisms of *Sporothrix* sp. exhibit significant complexity and intra- and interspecies variability, depending on the host’s immune response [55].

It has been noted that fungal phytopathogens have lower oxidative stress resistance, specifically to H_2_O_2_, compared to human fungal pathogens [56]. This supports the idea that species within the *Sporothrix* genus exhibit variability in virulence and resistance to oxidative stress from different types of ROS [27]. Mario et al. [26] compared oxidative stress indicators using a catalase assay in tissue samples from mice infected with *S. brasiliensis* and *S. schenckii*. Their results highlight the importance of magnesium levels as a protector against oxidative stress and for maintaining the proper function of immune cells. They reported that mice infected with *S. brasiliensis* exhibited greater damage (splenomegaly) and a significant decrease in catalase activity. Furthermore, substantial heterogeneity in virulence profiles among different species of the genus *Sporothrix* has been demonstrated [34].

Shi et al. [57] demonstrated that different *Sporothrix* species, except *S. brasiliensis*, induced the differentiation of normal density neutrophils (NDNs) into low-density neutrophils (LDNs) in patients with sporothrichosis. This was associated with increased infection severity, as phagocytosis and ROS production were decreased. However, differentiation of NDNs into LDNs could also be related to their mobilization from the bone marrow to the peripheral blood to respond to the chemotactic processes of these cells during infection. Furthermore, NDNs showed higher resting ROS levels than low-density neutrophils. Therefore, evasion mechanisms by *Sporothrix* sp. must play a role in the immune response [57].

## 3. Proteomic Insights into the Response to Oxidative Stress and Its Impact on Virulence

Many studies conducted to increase our understanding of sporotrichosis show different virulence degrees related to different *Sporothrix* spp. In that sense, Arrillaga-Moncrieff et al. [54] implemented murine sporotrichosis models with strains belonging to the species *S. albicans, S. globosa, S. mexicana, S. brasiliensis,* and *S. schenckii*. They found that *S. brasiliensis* and *S. schenckii* strains showed higher virulence and lethality. Such differences in virulence have been associated with differences in their expression of proteins. For example, the humoral response in mice recognized immunogenic molecules of 60 and 110 kDa within the exoantigens of the most virulent strains of *S. brasiliensis, S. globosa*, and *S. schenckii* [34]. The differential expression of surface proteins with adhesion capacity has also been related to higher virulence. In murine models, the *Sporothrix* strains with a lower virulence have poor adhesion to fibronectin and laminin, constitutive proteins of the extracellular matrix [58]. As a relevant fact, the main adhesin described in the cell surface of *S. schenckii*, Gp70, can mediate the adhesion of yeasts to the dermic extracellular matrix and modulate the immune response of the host, demonstrating its important role as both an antigen and a virulence factor [59,60]. Nevertheless, the differential expression of immunogen and high-adhesion-capacity proteins is not the only relevant strain feature related to sporotrichosis pathogenesis. The differential expression of proteins related to the evasion of the immune mechanisms of the host (detoxification or molecular patterns associated with pathogens), as well as the expression of multirole moonlighting proteins located in a different place not associated with their primary tasks, must be considered [61]. Therefore, whole-protein-profile studies among different *Sporothrix* spp. could explain the virulence differences within the same genus, and these differences could be associated with distinct mechanisms used to evade the immune response. According to [62], through ultra-resolution mass spectrometry analysis in proteinic extracts, it was found that only *S. brasiliensis* strains, compared to *S. schenckii* strains, expressed proteins associated with carbohydrate metabolism (glyceraldehyde 3-phosphate dehydrogenase and Acetyl-Coa hydrolase), detoxifying proteins (superoxide dismutase), and cell wall restructuration proteins (extracellular CW glucanase). Silva-Bailao et al. [63] conducted a comparative proteomic study and demonstrated that *S. brasiliensis, S. schenckii*, and *S. globosa* strains differentially expressed enzymes associated with glycolytic, tricarboxylic acids, and phosphate pentose pathways, chitin synthesis, CW remodeling, lipid and amino acid metabolism, and oxidative stress response proteins, such as CAT, SOD, peroxidase (Px), glutathione reductase (GR), and glutathione S-transferase (GST), all directly related to oxidative stress response mechanisms present in the fungus [63].

Oxidative stress generated by phagocytic cells such as monocytes and macrophages produces ROS/RNS to clear fungal invasion [49]. ROS have been used to simulate the harsh conditions that fungi face once inside the host organism. Evidence suggests that *S. schenckii* yeast activates mechanisms to detoxify or eliminate the harmful reactive species when exposed to H_2_O_2_. The fungus increases the expression of proteins related to oxidative stress, such as mitochondrial peroxiredoxin-1 (Prx1) and a 70 kDa heat shock protein (Hsp70) [41]. Interestingly, Prx1 expression was directly proportional to H_2_O_2_ concentration, and *HSP70* expression levels showed a maximum at 200 mM of H_2_O_2_ but its expression was diminished at higher concentrations [41].

*S. schenckii* CWPs after exposure to menadione, a different ROS, have also been studied. The results indicate that the fungus produced enzymes associated with oxidative stress (Trx and SOD), enzymes related to metabolic processes (glucose hydrolase, fructose biphosphate aldolase, citrate synthase and trehalase), and CW remodeling or organization proteins (β-1,3-endoglucanase EglC and chitinase) [23]. The proteomic analysis of *S. schenckii* exposed to H_2_O_2_ found that 28 CWPs modulated its response to oxidative stress. Central proteins associated with detoxifying mechanisms (Prx, SOD, and Trx), CW remodeling (β-1,3-endoglunase, β-glucosidase, β-1,3-glucanosil transferase, glycoside hydrolase, and Crf1 glycosidase), metabolic processes (glyceraldehyde 3 phosphate dehydrogenase, phosphoglycerate synthase, and citrate synthase), and trehalose synthesis (trehalose 6 phosphate synthase, trehalose synthase) remarkably modulated their expression levels [24]. According to the abovementioned studies, the differential expression of proteins associated with oxidative stress plays a key role in *S. schenckii* virulence. Therefore, elucidation of the mechanisms used to activate or inhibit these virulence factors could pave the way for designing new treatment strategies against sporotrichosis infection.

The expression of three different catalases, CAT1, CAT2, and CAT3, with predicted molecular weights of 57.6, 56.2, and 81.4 kDa, respectively, was demonstrated in the yeast morphotype of *S. schenckii* exposed to increasing concentrations of H_2_O_2_ [14]. Remarkably, the expression levels of CAT1 were higher than CAT2 and CAT3 levels, suggesting that CAT1 could play a key pathogenic role in evading the host immune response, therefore being a potential therapeutic target in sporotrichosis [15]. These authors remarked the relevance of the antioxidant mechanisms of the *Sporothrix* genus and shed light on several questions: What kind of regulation do the catalases suffer? Are the structural features in the catalases within the *Sporothrix* genus responsible for the catalytic activity differences? Or could these catalases be sensitive to new inhibitory compounds? Further studies are needed to better understand the regulatory mechanisms affecting catalases in the *Sporothrix* genus. While structural features may contribute to differences in catalytic activity, the exact relationship remains unclear. Additionally, it is important to investigate whether these catalases are susceptible to new inhibitory compounds, which could inform potential therapeutic strategies. In the context of potential therapeutic targets represented by virulence factors associated with the *S. schenckii* oxidative stress response, Sierra-Campos et al. [64] carried out assays to inhibit the growth of *S. schenckii* yeast challenged with different concentrations of *Moringa oleífera* extracts. This plant contains more than 200 bioactive molecules that potentially control fungal diseases. Their study found that the minimal inhibitory concentration and media lethal concentration were in the range of 0.5–8 µg/µL and 1–16 µg/µL, respectively. Additionally, their work also demonstrated that the bioactive molecules glucosinalbin and glucomoringin, present in *M. oleifera*, modified the kinetic parameters Km and Vmax of catalase, which made them promising candidates for inhibiting this enzyme and the oxidative stress response in *S. schenckii* [64].

## 4. Transcriptional Responses to ROS in *S. schenckii*

Opportunistic fungi such as *S. schenckii* enter the host through wounds and must resist the onslaught of the innate immune system defenses. The host immune system activates the phagolysosomal pathway to destroy fungal cells by limiting nutrients, generating various lytic enzymes and ROS that damage pathogen cells through the so-called respiratory burst mediated by NADPH oxidase [65]. Specific concentrations of exogenous ROS are known to be capable of producing damage to DNA, lipids, and proteins and ultimately causing programmed cell death in pathogens [65]. As a counterpart, pathogenic fungi activate the machinery necessary for detoxification of these oxygen species through signaling pathways that turn on transcription factors, which regulate the production of antioxidant enzymes [66]. The most well-known oxidative stress response signaling pathway in yeasts such as *Saccharomyces cerevisiae* and the pathogens *Candida albicans*, *Cryptococcus neoformans,* or *Paracoccidioides brasiliensis* is the one mediated by the MAP kinase HOG1, which activates transcription factors such as Atf1, Yap1, and Skn7 that turn on the genes encoding detoxifying proteins such as CAT, GPx, or SOD [67]. The HOG1-mediated signaling pathway is one of the most conserved in eukaryotes since it intervenes in ROS signaling and osmotic stress [68]. In the dimorphic fungus *C. albicans*, the Hog1 MAPK (SAPK) is phosphorylated during exposure to oxidative stress of H_2_O_2_ (>2 mM) [69], as well as diverse oxidants, including the oxidative burst of phagocytes [70].

A study in *S. schenckii* and *S. brasiliensis* showed that a more virulent *S. brasiliensis* strain (MYA4823) is more resistant to oxidative stress compounds such as menadione and H_2_O_2_ compared to *S. schenckii* strains (MYA4820 and MYA4821) and a less virulent *S. brasiliensis* strain (MYA4824). Additionally, the study showed that both *S. schenckii* and *S. brasiliensis* genomes contain an AP1-like transcription factor (SsAP1 and SbAP1) with no significant differences in their sequences. These strains also contain similar genes implicated in oxidative stress responses regulated by the AP1 transcription factor, including CAT, GPx, thioredoxin peroxidases (TrxP), and SOD [27]. Furthermore, they showed that SOD activity did not differ in the virulent *S. brasiliensis* strain from that in *S. schenckii* strains. Finally, to understand the differences in ROS resistance, they analyzed the SsHog1 and SbHog1 genomic sequences encoding the Hog1 MAPK (SAPK), finding that SbHog1 from *S. brasiliensis* is missing 14 conserved amino acids within the N-terminal region compared to SsHog1 from *S. schenckii* and other fungi [27]. Another transcription factor preventing intracellular ROS accumulation during hyphal growth studied in *C. albicans*, *Candida glabrata*, and *S. cerevisiae* is Skn7 [71,72,73]. In the yeast *S. cerevisiae*, a null mutant in Skn7 resulted in hypersensitivity to peroxides and menadione [73]. Similarly, in *C. glabrata*, a mutant lacking *SKN7* was hypersensitive to H_2_O_2_, and the induction of its regulated genes, thioredoxins Trx2, Trr1, peroxidase (Tsa1), and catalase (CAT1), was abolished or delayed. Moreover, the *SKN7* null mutant displayed attenuated virulence in a mouse model [72]. The transcription factor and its regulatory genes have not been studied in *Sporothrix* sp. Therefore, studying the involvement of Skn7 in ROS detoxification and virulence is necessary to understand the regulation of genes involved in ROS resistance and detoxification during oxidative stress.

## 5. Future Directions

Despite advances in understanding the mechanisms that allow *S. schenckii* to cope with oxidative stress and evade the host’s immune response, significant knowledge gaps remain, opening up several avenues for future research. An interesting aspect that also emerges is that the different morphotypes of *S. schenckii* interact variably with macrophage receptors, leading to distinct immune responses. For example, opsonized conidia and yeast forms are recognized differently by THP-1 macrophages, resulting in varied production of ROS and cytokines [49]. This suggests that *S. schenckii* not only adapts to oxidative stress but may also influence the host’s oxidative response mechanisms.

These findings highlight the importance of thoroughly characterizing the molecular pathways involved in ROS detoxification and their regulation during host–pathogen interactions. The use of more physiologically relevant in vitro and in vivo models will allow for a better approximation of infection dynamics. Additionally, comparing these mechanisms across different species within the *S. schenckii* complex may reveal species-specific virulence determinants. The identification of novel factors involved in immune evasion and intracellular survival could also provide therapeutic targets for the development of more effective anti-fungal strategies, including combination therapies or the rational design of vaccines in clinical and zoonotic contexts.

In addition, the presence of LDNs with reduced oxidative capacity in sporotrichosis suggests that *S. schenckii* may face a less hostile phagocytic environment, potentially facilitating its survival and persistence. Investigating how *S. schenckii* responds to oxidative stress under these conditions could uncover specific mechanisms of adaptation and immune evasion. This line of research offers valuable insights into fungal persistence within the host and may guide the development of therapeutic strategies aimed at enhancing phagocytic function. Moreover, the accumulation of LDNs might not merely reflect a dysfunctional immune phenotype but rather an active recruitment process driven by host- or fungus-derived chemotactic signals. Exploring whether *S. schenckii* can influence LDN differentiation or mobilization from bone marrow could reveal a novel immune evasion strategy in sporotrichosis. A deeper understanding of these interactions would help integrate the pathogen’s oxidative stress response with the immunological context of the host, contributing to a more comprehensive view of the fungus–phagocyte interplay.

## 6. Conclusions

**Figure 2:**
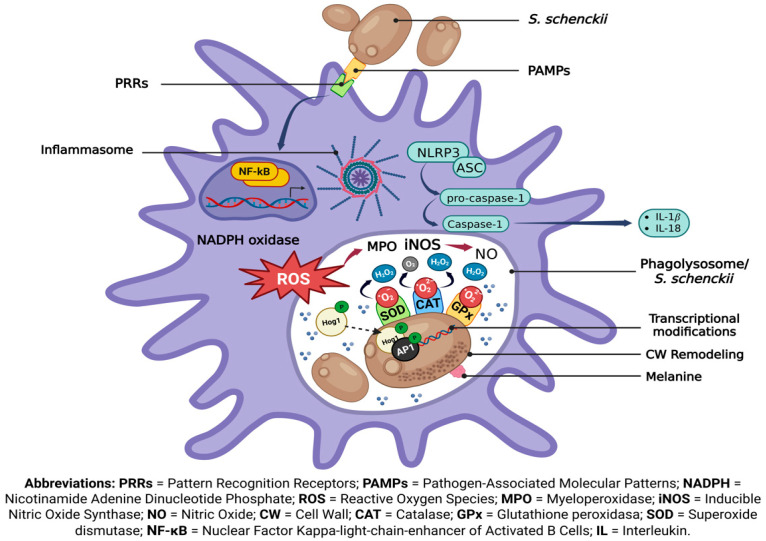
The interaction between *S. schenckii* and phagocytic cells. Following the recognition of PAMPs by PRRs on the phagocytic cell, the phagocytosis of *S. schenckii* triggers oxidative stress pathways mediated by NADPH oxidase and MPO, leading to the production of ROS. Nuclear translocation of NF-κB is required for the transcription of NLRP3 (NOD-like receptor family pyrin domain containing 3), which is activated by “danger signals” due to fungal infection and assembles the inflammasome with the participation of the ASC protein, which facilitates the activation of caspase-1. This promotes the maturation and secretion of the inflammatory cytokines IL-1β and IL-18. In response, *S. schenckii* produces melanin, CWPs (some of which are involved in CW remodeling), and antioxidant enzymes such as SOD, CAT, and GPx (regulated by the transcription factor AP-1) as they enable the fungus to adapt and survive against ROS generated within the phagolysosome.

The generation of ROS by phagocytic cells such as macrophages and neutrophils is one of the host’s primary defense mechanisms. However, *S. schenckii* has developed complex strategies to detect, respond, and resist adverse conditions, ensuring its survival within the host’s hostile environment. Figure 2 summarizes the mechanisms involved in *S. schenckii* interaction with the phagocyte and its response to oxidative stress. Understanding these processes of fungal adaptation could reveal novel therapeutic targets by identifying vulnerable critical points for pharmacological interventions. Furthermore, novel clinical strategies for managing fungal infection resistance could be designed and improved by elucidating the molecular mechanisms governing oxidative stress resistance.

## Figures and Tables

**Table 1:** Reactive oxygen species (ROS) interacting with *S. schenckii* during sporotrichosis.

ROS	Function	Cell Involved and/or Enzyme	Reference
Superoxide (O_2_)	First ROS generated in the respiratory burst. Toxic to pathogens and is converted into other ROS.	Neutrophils and macrophages via NADPH oxidase.	[40]
Hydrogen peroxide (H_2_O_2_)	Generated from superoxide, participates in pathogen destruction and can form hydroxyl radicals.	Neutrophils and macrophages via superoxide dismutase (SOD).	[41]
Hydroxyl radical (OH)	Highly reactive ROS that damages lipids, proteins, and DNA of the fungus.	Formed by the Fenton reaction (Fe^2+^ + H_2_O_2_) in neutrophils.	[39]
Hypochlorous acid (HOCl)	Potent microbicidal agent that oxidizes proteins and lipids in pathogens.	Neutrophils produced by the enzyme myeloperoxidase (MPO).	[42]
Nitric oxide (NO)	Has antimicrobial properties and combines with superoxide to form peroxynitrite.	Activated macrophages via inducible nitric oxide synthase (iNOS).	[43]
Peroxynitrite (ONOO)	Oxidizes essential components of the pathogen, such as lipids, proteins, and nucleic acids.	Formed by the interaction of O_2_⁻ and NO in activated macrophages.	[44]
Singlet oxygen species (^1^O_2_)	Direct oxidative damage to pathogen biomolecules.	Neutrophils during the respiratory burst.	[45]

**Table 2:** Evasion mechanisms of *S. schenckii* against ROS.

Evasion Mechanism	Function	Reference
Melanin production	Neutralizes ROS such as O_2_⁻ and H_2_O_2_, acting as a natural antioxidant. Protects the fungus from oxidative damage and enhances resistance to phagocytosis.	[51]
Antioxidant enzymes		
Superoxide dismutase (SOD)	Converts O_2_*⁻* into H_2_O_2_, reducing direct oxidative damage.	[24]
Catalase (CAT)	Decomposes H_2_O_2_ into water and oxygen, neutralizing its toxicity.	[14]
Glutathione peroxidase (GPx)	Reduces H_2_O_2_ and other organic peroxides via the glutathione system.	[52]
Cell wall (CW)		
CW remodeling	The composition of β-glucans, chitin, and proteins can be altered to resist ROS and adapt to oxidative stress.	[23,24,53]
Heat shock protein (HSP) production	These proteins protect fungal proteins from denaturation and oxidative damage.	[23,24,41]

## Data Availability

No new data were created or analyzed in this study. Data sharing is not applicable to this article.
